# Partial segmental thrombosis of the corpus cavernosum: a case report and review of the literature

**DOI:** 10.1093/jscr/rjag598

**Published:** 2026-07-21

**Authors:** Abdulrahman Aljofan, Mohammed Alzahrani, Mohammed Abunayan, Abdulrahman Aldouir, Abdelmoniem Eltiraif

**Affiliations:** College of Medicine, King Saud University, PO Box 2925, Riyadh 11461, Saudi Arabia; Department of Urology, King Saud University Medical City, PO Box 36175, Riyadh 11419, Saudi Arabia; Department of Urology, King Saud University Medical City, PO Box 36175, Riyadh 11419, Saudi Arabia; College of Medicine, King Saud University, PO Box 2925, Riyadh 11461, Saudi Arabia; Department of Urology, King Saud University Medical City, PO Box 36175, Riyadh 11419, Saudi Arabia

**Keywords:** partial segmental thrombosis, corpus cavernosum, penile pain, anticoagulation

## Abstract

Partial segmental thrombosis of the corpus cavernosum (PSTCC) is a rare urological condition presenting as localized perineal pain and asymmetric corporal swelling. We report a 27-year-old male with subacute penile pain and decreased erectile rigidity following a prolonged erection. The patient had a contrast-enhanced computed tomography (CT) scan showing asymmetrical enlargement with thrombus of the left corpus cavernosum. He was managed conservatively with rivaroxaban and referred for thrombophilia testing, which returned negative. While magnetic resonance imaging is the gold standard for diagnosing PSTCC, this case demonstrates that contrast-enhanced CT can serve as an effective diagnostic tool in the acute setting, supporting a non-invasive diagnostic and treatment approach.

## Introduction

Partial segmental thrombosis of the corpus cavernosum (PSTCC) is an extremely rare urological condition that predominantly affects young men [1]. The condition was initially described as ‘partial priapism’ by Gottesman *et al*. in 1976; it is described as the same entity as partial thrombosis of corpus cavernosum [2]. Priapism, characterized by a persistent and painful penile erection, must be distinguished from isolated thrombosis of corpus cavernosum [3]. PSTCC involves thrombosis of the proximal segment of the corpus cavernosum, in contrast to classical ischemic priapism, which primarily affects the distal portion [4]. Although the underlying mechanism has not been definitively established, several precipitating factors have been proposed, including blunt perineal trauma, hematological disorders, recreational drug use such as cocaine or marijuana, and pharmacologic agents including tamsulosin and sildenafil. Repetitive mechanical stress during sexual intercourse or prolonged cycling may also contribute to thrombus formation [5, 6]. Clinical manifestations commonly include a tender, palpable perineal mass accompanied by varying degrees of erectile dysfunction [4, 7]. Furthermore, imaging plays a central role in diagnosis. Ultrasonography typically shows asymmetric enlargement of the involved corpus cavernosum containing heterogeneous material and lacking vascularization on color Doppler study. Correspondingly, magnetic resonance imaging (MRI) reveals cavernosal expansion with signal alterations compatible with thrombus formation [7]. Management is preferably conservative and typically includes systemic anticoagulation and adequate analgesia. Surgical intervention should be considered only in cases of persistent or refractory pain, development of erectile dysfunction, or recurrence despite appropriate medical therapy [7, 8].

## Case presentation

A 27-year-old male presented to the emergency department with a 1-month history of penile pain radiating to the left thigh. The symptoms began following an episode of prolonged erection lasting ˃4 h, which resolved spontaneously without medical intervention. Subsequently, the patient experienced recurrent episodes of penile pain occurring in the flaccid state, with exacerbation during erection. He also reported a recent decline in erectile rigidity. His past medical history was notable for asthma and a history of substance abuse; otherwise, it was unremarkable, with no prior surgical history. The patient denied any history of sexual trauma, dysuria, or urethral discharge. He also reported no excessive cycling, recent masturbation-related trauma, or long-distance travel.

On physical examination, the patient appeared to be in discomfort. The penis was flaccid, with no evidence of erection. There was marked tenderness along the left penile shaft extending to the perineum and left gluteal region. No erythema, fluctuance, crepitus, or signs of infection were observed. Both testes were normal in size and position, without swelling or tenderness.

An enhanced computed tomography (CT) scan of the abdomen and pelvis was performed (Fig. 1), revealing asymmetrical enlargement of the left corpus cavernosum with internal peripherally enhancing hypodensity, consistent with segmental thrombosis of the corpus cavernosum.

**Figure 1:**
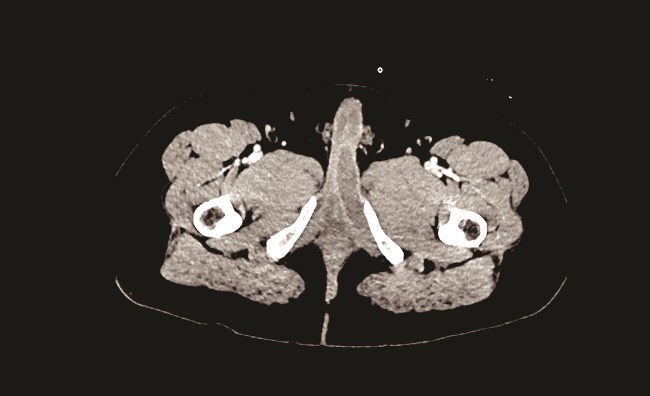
CT abdomen and pelvis with contrast (axial view) asymmetrical enlargement of the left corpus cavernosum with internal peripherally enhancing hypodensity, suspicious for partial thrombosis.

A conservative management was initiated, consisting of anticoagulation therapy (Rivaroxaban 20 mg once daily) for a planned duration of 3–6 months. He was also referred to hematology for thrombophilia evaluation, which was negative. Scheduled for urological follow-up at 3 months. At the time of manuscript preparation, he had not yet returned for clinical reassessment.

## Discussion

PSTCC is an uncommon condition primarily described in case reports and small series. Earlier literature referred to this entity as ‘partial priapism,’ but more recent publications recognize it as a localized cavernosal thrombotic process distinct from classical ischemic priapism [1, 7]. Unlike diffuse rigid erection seen in classical priapism, PSTCC typically presents with localized proximal corporal pain and asymmetric enlargement without complete tumescence [7].

Pain is the primary symptom, and erectile dysfunction, when present, is variable in onset and severity [1, 9]. Ilicki *et al*. noted that erectile dysfunction resulting from PSTCC often improves with conservative therapy [1], while Christodoulidou *et al*. suggested it may evolve rather than occur immediately at presentation [9]. In our patient, the decline in erectile rigidity following a prolonged erection supports a subacute thrombotic process rather than classical ischemic priapism.

The etiology of PSTCC is frequently idiopathic [1, 10]. A proposed ‘two-hit’ hypothesis suggests that localized thrombosis of the intracavernosal septum occurs when a hemodynamic trigger acts on an anatomically predisposed site [7]. In our patient, a prolonged erection exceeding 4 h likely induced sufficient venous stasis to precipitate thrombosis, in the absence of identifiable trauma or thrombophilia. Sildenafil exposure and physical exertion have also been cited as potential triggers in the literature [5, 11].

Imaging is central to both diagnosis and exclusion of alternative pathology. MRI remains the gold standard, offering superior soft-tissue characterization and thrombus assessment [7, 12]. However, contrast-enhanced CT can effectively identify the hallmark findings of asymmetric cavernosal enlargement with internal hypodensity and peripheral enhancement. Patel *et al*. previously reported CT findings consistent with unilateral proximal cavernosal enlargement suggestive of thrombosis, subsequently confirmed on MRI and follow-up imaging [10]. Our case similarly demonstrates that CT alone can provide sufficient diagnostic confidence to proceed with conservative management, particularly when MRI is unavailable. We recognize the absence of confirmatory MRI as a limitation, as it would have allowed more complete anatomical assessment.

Management has evolved away from aggressive surgical intervention—including direct exploration or shunt placement, often performed secondary to incomplete diagnostic evaluation—toward conservative strategies [1, 7, 13]. Anticoagulation with a DOAC—specifically rivaroxaban—combined with analgesia has proven effective in recent reports and was used in our patient for 3–6 months [1, 5, 14]. Routine thrombophilia screening is not recommended, but targeted evaluation is warranted in young patients without a clear precipitant or with recurrent episodes [1, 15]. Our patient’s thrombophilia workup returned negative.

## Conclusion

PSTCC is a rare but important differential diagnosis for young men presenting with localized perineal or penile pain. While the ‘two-hit’ hypothesis involving venous stasis and anatomical predisposition remains a likely mechanism, many cases present without clear mechanical trauma. Although MRI is the preferred diagnostic modality, our case demonstrates that contrast-enhanced CT is a viable tool for identifying characteristic thrombotic changes when MRI is unavailable. Current evidence strongly supports a conservative management strategy using anticoagulation and analgesia, which typically results in symptomatic resolution and prevents unnecessary surgical intervention.

## References

[1] Ilicki J, Krauss W, Andersson SO. Partial segmental thrombosis of the corpus cavernosum: a case report and a review of the literature. Urology 2012;79:708–12. 10.1016/j.urology.2011.11.032 22386425

[2] Gottesman JE . Recurrent partial priapism. Urology 1976;7:519–20. 10.1016/0090-4295(76)90195-3 1274013

[3] Goeman L, Joniau S, Oyen R et al. Idiopathic partial thrombosis of the corpus cavernosum: conservative management is effective and possible. Eur Urol 2003;44:119–23. 10.1016/s0302-2838(03)00217-3 12814686

[4] Hulth M, Albersen M, Fode M et al. Idiopathic partial thrombosis of the corpus cavernosum: aetiology, diagnosis and treatment. Scand J Urol 2013;47:163–8. 10.3109/00365599.2012.698305 22762201

[5] Danchi HG, Kay C, Hole SR et al. Partial segmental thrombosis of the corpus cavernosum associated with inappropriate sildenafil use. Cureus 2024;16:e53462. 10.7759/cureus.53462 38435230 PMC10909401

[6] Baraças CA, Pinto J, Tavares MC. Partial thrombosis of the corpus cavernosum - a malignancy mimicker. BJR Case Rep 2021;8:20210085. 10.1259/bjrcr.20210085 35136638 PMC8803217

[7] Weyne E, Schillebeeckx C, Jamaer C et al. Idiopathic partial thrombosis (IPT) of the corpus cavernosum: a hypothesis-generating case series and review of the literature. J Sex Med 2015;12:2118–25. 10.1111/jsm.13036 26553854

[8] Kammo S, Hussain B, Dabaja A. Partial segmental thrombosis of the corpus cavernosum: a comprehensive literature review. Ann Reprod Med Treat 2025;8:1–7. 10.47739/reproductivemedicine

[9] Christodoulidou M, Parnham A, Ramachandran N et al. Partial segmental thrombosis of the corpus cavernosum presenting with perineal pain. BMJ Case Rep 2016;2016:bcr2016216263. 10.1136/bcr-2016-217748 PMC5129098 27879305

[10] Patel RP, Mucksavage P, Ramchandani P et al. Idiopathic partial thrombosis of the corpus cavernosum. Urology 2010;76:1373–4. 10.1016/j.urology.2009.12.058 20394978

[11] Rybár L, Hesko P, Kollárik B et al. Partial segmental thrombosis of the corpus cavernosum post prolonged run and MRI changes during follow-up: a case report. Urol Case Rep 2023;51:102615. 10.1016/j.eucr.2023.102615 38046257 PMC10689872

[12] Kimball DA, Yuh WT, Farner RM. MR diagnosis of penile thrombosis. J Comput Assist Tomogr 1988;12:604–7. 10.1097/00004728-198807000-00012 3392260

[13] Lewis JH, Javidan J, Keoleian CM et al. Management of partial segmental priapism. Urology 2001;57:169. 10.1016/S0090-4295(00)00851-7 11164175

[14] Baaklini G, Reed A, Tafti D et al. Corpus cavernosum thrombosis case report. Urol Case Rep 2021;36:101593. 33659186 10.1016/j.eucr.2021.101593 PMC7890131

[15] Senthilkumaran S, Sampath S, Miller SW et al. Partial segmental thrombosis of the corpus cavernosum following Russell’s viper envenomation. Toxicon 2023;234:107284. 10.1016/j.toxicon.2023.107284 37683700

